# Supplementary material for: Observational pilot using a Data to Care intervention strategy to promote HCV re-engagement and cure for persons with HIV/HCV co-infection who are out of care
Source: BMC Health Serv Res. 2025 Jan 25;25:140. doi: 10.1186/s12913-025-12307-6 (PMC11763122; doi:10.1186/s12913-025-12307-6)
Supplement: Supplementary file 1 — Supplementary Material 1. [file 12913_2025_12307_MOESM1_ESM.docx]

**APPENDIX:**

*Protocol for DIS Outreach Efforts*

**Patient contact**

1. Call the patient using the contact information provided on OOC list and/or the additional contact information from the provider
2. If the call goes to voicemail, leave the following message:

“Hello, my name is **[DIS name]** and I am calling from the Connecticut Department of Public Health. I am calling for **[patient name]** and I would like to ask him/her a few questions regarding their health. If you would please call me back at **[DIS phone number]** that would be great. Again, my name is **[DIS name]**, I am calling from the Connecticut Department of Public Health, and my number is **[DIS phone number]**. Thank you”.

1. Try calling three times leaving three voice messages. After the third time, send a letter to the patient (see attachment for letter template). Wait a week for a reply; if none, then mark as *Loss to Follow-up* on the DIS Tracking Log under the *Completion Outcomes* column and move on.
2. If the patient does answer, introduce yourself and verify their identity by having them tell you what their DOB is. Next, explain the purpose of the call and of the project stating you are trying to connect them with their provider to treat/cure their HCV. Let them know you will help them make their appointments and you will be following them throughout the treatment process (ultimately helping them until everything is complete).
   1. Collect information on the days and times that work best for them in order to help make their provider appointments.
3. Continue additional patient contact based on DPH training (i.e. field visits, test messages, social media messages, email, etc.) if patient does not respond within 30 days.

**Make the appointment and continued follow-up**

1. Call the provider and make the appointment using the day and time information from the patient
2. Contact the patient and inform them of the appointment day and time. Advise them that you will call them the day before or the morning of (whatever is more convenient) to remind them.
3. Call the patient to remind them of their appointment. If the call goes to voicemail leave the following message: “Hello **[patient name]**, I am calling to remind you about your appointment today/tomorrow at **[appointment time]**. If you have any questions, please call **[DIS number]**. Thank you”.
4. Once the appointment day and time has passed, call the patient to see if they made it in:
   1. If so, have DIS manager record it on the DIS Tracking Log
   2. If not, follow-up with the patient again and ask them what kept them from seeing their provider. Use this information to avoid any barriers preventing them from going again. Once this is done, begin Step 4 again.
5. Continue following-up with the patient and making appointments until one of the completion outcomes in the Tracking Log is achieved.
